# New synonyms in the highly diverse caddisfly genus *Smicridea* (Trichoptera, Hydropsychidae)

**DOI:** 10.3897/zookeys.637.10148

**Published:** 2016-11-28

**Authors:** Ernesto Rázuri-Gonzales, Ralph W. Holzenthal

**Affiliations:** 1 Department of Entomology, University of Minnesota, 219 Hodson Hall, 1980 Folwell Avenue, St. Paul, Minnesota 55108, USA; 2 Departamento de Entomología, Museo de Historia Natural, Universidad Nacional Mayor de San Marcos, Apartado 14-0434, Lima 14, Peru

**Keywords:** Synonymy, New combination, Neotropics, Nearctic, Trichoptera

## Abstract

In this paper, Smicridea (Rhyacophylax) repula Oláh & Johanson, 2012 is synonymized with Smicridea (Rhyacophylax) lobata (Ulmer, 1909), and the species *Leptonema islamarga* Botosaneanu, 2002 is transferred to Smicridea (Rhyacophylax) as a synonym of *Smicridea lobata*. Additionally, we present more detailed illustrations of the male genitalia of Smicridea (Rhyacophylax) lobata and Smicridea (Rhyacophylax) signata (Banks, 1903), and include notes on their distributions to aid in the identification of these two, often-confused species.

## Introduction

The genus *Smicridea* was established by McLachlan (1871) to include the species *Smicridea fasciatella* from Texas. The genus now contains 232 species, making it, by far, the largest Hydropsychidae genus in the Western Hemisphere. The genus occurs from the southwestern USA, through Mexico, Central America, the Caribbean, and all of South America. It is divided into two subgenera: the nominotypical *Smicridea* (130 species) and *Rhyacophylax* Müller 1879 (102 species); the subgenera are based mainly on differences in the wing venation (Flint 1974a).

In the subgenus Rhyacophylax, the *signata* group of Flint (1974a) is characterized by a fixed, tongue-like, ventromesal process on the apex of the phallus, and the presence of a lobe with spinose processes developed in various numbers and positions, arising from the ventrolateral margin of the tenth tergum. Sixteen species distributed from northern South America, throughout Central America, into southwestern USA (Arizona, Colorado, New Mexico, Texas, and Utah) are included in this group (Table 1).

**Table 1.** Smicridea (Rhyacophylax) signata species group.

Species name	Author	Distribution
Smicridea (Rhyacophylax) arizonensis	Flint 1974b	Mexico, USA
Smicridea (Rhyacophylax) bidactyla	Flint and Reyes 1991	Ecuador, Peru, Venezuela
Smicridea (Rhyacophylax) bifurcata	Flint 1974a	Costa Rica, Honduras
Smicridea (Rhyacophylax) fogasa	Oláh and Johanson 2012	Ecuador
Smicridea (Rhyacophylax) hajla	Oláh and Johanson 2012	Ecuador
Smicridea (Rhyacophylax) inarmata	Flint 1974b	Mexico
Smicridea (Rhyacophylax) kampoka	Oláh and Johanson 2012	Peru
Smicridea (Rhyacophylax) leloga	Oláh and Johanson 2012	Peru
Smicridea (Rhyacophylax) lobata	(Ulmer 1909)	Costa Rica, El Salvador, Guatemala, Honduras, Mexico, Nicaragua, Panama, Venezuela
Smicridea (Rhyacophylax) nemorosa	Holzenthal and Blahnik 1995	Costa Rica
Smicridea (Rhyacophylax) nemtompa	Oláh and Johanson 2012	Ecuador, Peru
Smicridea (Rhyacophylax) pseudolobata	Flint 1978	Brazil, Suriname
Smicridea (Rhyacophylax) salta	Flint 1974b	Mexico
Smicridea (Rhyacophylax) signata	(Banks 1903)	Guatemala, Mexico, USA
Smicridea (Rhyacophylax) singri	Holzenthal and Blahnik 1995	Costa Rica
Smicridea (Rhyacophylax) tavola	Oláh and Johanson 2012	Ecuador

*Smicridea lobata* (Ulmer 1909, in Ulmer and Thienemann 1909) was described from Las Trincheras (Venezuela), from a single male specimen preserved in alcohol. Ulmer mentioned that the forewing coloration of *Smicridea lobata* resembled that of *Smicridea columbiana* (Ulmer 1905), but he did not compare the genitalia of these two species or those of any of the species in the genus known at the time. Later, Flint (1974b) doubtfully recorded *Smicridea lobata* from Surinam. Even though he did not examine the type, he stated that the specimens he studied agreed with the illustration of the type of *Smicridea lobata* provided by Ulmer. Additionally, after carefully examining the type specimen of *Smicridea lobata*, Flint (1978) concluded that the species he referred to as *Smicridea lobata* in his earlier paper was actually a different species, which he described as *Smicridea pseudolobata*, due to differences in the tenth tergum and the phallus.

*Smicridea repula* Oláh & Johanson, 2012 was described from Los Tuxtlas area in the state of Veracruz (Mexico). The authors included this species in the *signata* group, stating that it was closely related to the species *Smicridea lobata* from Venezuela and *Smicridea nemtompa* Oláh & Johanson, 2012 from Ecuador and Peru. They indicated that their new species was easily distinguished from *Smicridea lobata* and *Smicridea nemtompa* by having a lateral wing-shaped process at the mid-length of the phallus.

*Leptonema islamarga* Botosaneanu, 2002, in Botosaneanu and Viloria 2002, was described from Isla Margarita, Venezuela, and was placed in the *Leptonema davisi* group of Flint, McAlpine and Ross 1987, based on characters of the male genitalia.

The species *Smicridea signata* (Banks, 1903) was originally described as *Pellopsyche signata*, from Fort Collins, Colorado (USA). The description was based on characteristics of the body and wings, with no genitalic characters included (the type is a female). Later, Ross (1944) transferred the species, as *Rhyacophylax signatus*, to *Rhyacophylax*, a separate genus at the time. More recently, Flint (1974a) redescribed the species as Smicridea (Rhyacophylax) signata, and illustrated the male and female genitalia as well as some features of the larva.

**Figure 1–2.**
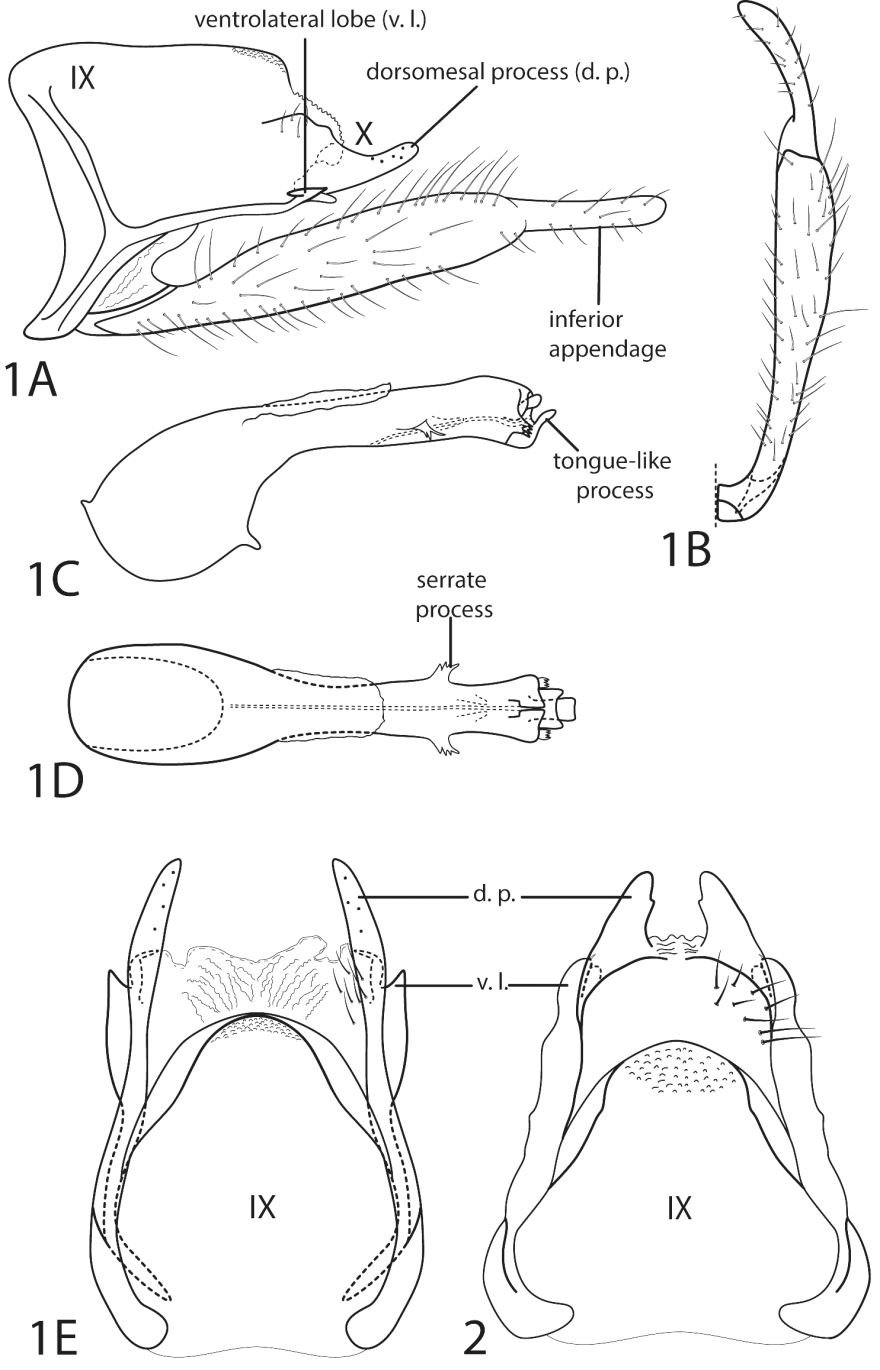
Smicridea (Rhyacophylax) lobata (Ulmer, 1909) and Smicridea (Rhyacophylax) signata Banks, 1903, male genitalia. **1A** Smicridea (Rhyacophylax) lobata segments IX and X, lateral **1B** Left inferior appendage, ventral **1C** Phallus, lateral **1D** Phallus, dorsal **1E** Segments IX and X, dorsal **2** Smicridea (Rhyacophylax) signata. Segments IX and X, dorsal. These illustrations were made from specimens of *Smicridea lobata* from Zulia and Sucre States, Venezuela, and specimens of *Smicridea signata* from Utah, USA.

We conclude that *Smicridea repula* and *Leptonema islamarga* are synonyms of *Smicridea lobata*, which is a separate species distinct from *Smicridea signata*, based on differences in the tenth tergum as well as in their distributions. Herein, we provide justification for these taxonomic changes as well as more detailed illustrations of *Smicridea lobata* from sites near the type locality (Fig. 1) and of *Smicridea signata* from Utah.

## Materials and methods

Specimens were examined with an Olympus SZH dissecting microscope (Olympus Corporation). The illustration of the male genitalia of *Smicridea lobata* was prepared from pencil sketches made with the aid of a drawing tube attached to an Olympus BX41 compound microscope. The pencil sketches were scanned and placed into an Adobe Illustrator CS6 (Adobe Systems, Inc.) document to serve as a template to create a vector graphic illustration. The careful tracing of the original image was accomplished by using a graphic tablet and pen (BAMBOO, Wacom Technology Co.).

We carefully examined specimens from the type series of *Smicridea repula* and *Leptonema islamarga*, borrowed from the Swedish Museum of Natural History (Stockholm, Sweden) and the Naturalis Biodiversity Center (Leiden, The Netherlands), respectively. Further, we examined material of *Smicridea signata* and *Smicridea lobata* identified by Dr Oliver Flint (National Museum of Natural History, Smithsonian Institution, Washington D.C.) and Dave Ruiter (Grants Pass, Oregon, USA) as well as material from the University of Minnesota Insect Collection (St. Paul, Minnesota, USA), and the female type of *Smicridea signata* from the Museum of Comparative Zoology, Harvard University, (Cambridge, Massachusetts, USA). The type of *Smicridea lobata* at the Natural History Museum of Denmark (Copenhagen, Denmark) could not be found (H. Enghoff, pers. comm.).

The material examined is deposited in the following institutions:

DRC
 Dave Ruiter, personal collection, Grants Pass, Oregon, USA 


NBC
Naturalis Biodiversity Center, Leiden, The Netherlands 


MCZ
Museum of Comparative Zoology, Harvard University, Cambridge, Massachusetts, USA 


NMNH
National Museum of Natural History, Smithsonian Institution, Washington D.C., USA 


NRM
Swedish Museum of Natural History, Stockholm, Sweden 


UMSP
University of Minnesota Insect Collection, St. Paul, Minnesota, USA 


### Material examined

Smicridea (Rhyacophylax) repula: **MEXICO: Veracruz**: Los Tuxtlas area, Río La Palma, near to the Estación de Biología Los Tuxtlas, 18°33.68'N, 95°02.94'W, 30 mao [meters above ocean], 26.VI.2006, light trap, leg. Espeland & Malm; 1 male holotype (NRM).

Smicridea (Rhyacophylax) signata: **USA: Colorado**: No further data; 1 female holotype (MCZ, type # 11513). **Arizona**: Clear Cr. Cmp., SE Camp Verde, 17.VI.1968, Flint & Menke; 1 male (NMNH). Greenlee County, light trap, Gila River near Duncan, 32°43.46'N, 109°06.01'W, ca 1120 m, 19.IV.2002, Blinn; 6 males, 6 females (DRC). **New Mexico**: Grant County, Gila River at Forks T13S R13W sec 8, 26.VII.2001, at light, Ruiter; 10 males, 6 females (DRC). **Texas**: Brewster County, Big Bend National Park, Terlingua Creek at Terlingua abaja [Terlingua baja], 29°15'N, 103°37.5'W, 680 m, 1.VI.1993; Gelhaus #607, Nelson & Koenig; 1 male (NMNH). **Utah**: San Juan County, San Juan River, RM 10.6, 37°15'N, 109°51'W, ca 1190 m, light trap, 23.V.2002, Hayden; 6 males, 76 females (DRC). **MEXICO: Chiapas**: Puente Arroyo Viejo, Rt. 200, km 141, 9.VI.1967, Flint & Ortiz; 10 males (NMNH). **Morelia**: Route 95, km 91, nr. Xochitepec, 1.VIII.1965, Flint; 1 male, 28 females (NMNH). Xochitepec, 12-14.VII.1965, Flint & Ortiz; 2 males, 12 females (NMNH). **Oaxaca**: Tehuantepec, 23.VII.1964, Spangler; 3 males, 19 females (NMNH). **San Luis de Potosí**: Palitla, 25.VI.1965, Flint; 3 males, 5 females (NMNH). **Veracruz**: Cordoba, 11-20.XI.1966, Lau, 2 males (NMNH). **GUATEMALA: Escuintla**: Escuintla, 10.VIII.1965, Spangler; 8 males, 10 females (NMNH).

Smicridea (Rhyacophylax) lobata: **MEXICO: Chiapas**: 7.8 mi E Pichucalco, 7.XII.1975, C. M. & O. S. Flint; 5 males (NMNH). Arriaga, 22.VIII.1965, Spangler; 5 males, 3 females (NMNH). Cascada Misol ha, 20 km S Palenque, 17-18.V.1981, C. M. & O. S. Flint; 3 males (NMNH). Puente Arroyo Viejo, nr. Mapastepec, 7.VIII.1966, Flint & Ortiz; 3 males (NMNH). Río Contento, 7 km N Ocosingo, 20.V.1981, C. M. & O. S. Flint; 1 male (NMNH). Río Tulija, 48 km S Palenque, 17.V.1981, C. M. & O. S. Flint; 8 males, 14 females (NMNH). **Oaxaca**: Dist. Choapan, Bethania, 31 km S San Juan Bautista Tuxtepec, 24.V.1981, C. M. & O. S. Flint; 6 males, 3 females (NMNH). Rancho San Pablo, 17 Km. E Tehuantepec, 23.V.1981, C. M. & O. S. Flint; 2 males (NMNH). Río Valle Nacional, Chiltepec, 25.V.1981, C. M. & O. S. Flint; 3 males, 1 female (NMNH). **San Luis de Potosí**: 1 mi W Tamazunchale, 11.VIII.1972, at black light, G. F. & S. Hevel; 3 males, 2 females (NMNH). **Veracruz**: Barranca de Metlac, Fortín de las Flores, 4.XII.1975, C. M. & O. S. Flint; 3 males, 5 females (NMNH). Barranca de Metlac, 6 km W Fortín, 1.V.1981, C. M & O. S. Flint; 1 male, 1 female (NMNH). Cuitlahuac, 10-12.VIII.1964, Spangler; 1 male (NMNH). Same, but 24-27.VII.1965, Flint & Ortiz; 1 male (NMNH). La Palma, nr. Sontecomapan, 5.XII.1975. C. M. & O. S. Flint; 9 males (NMNH). Los Tuxtlas area, Los Tuxtlas Biological Station, 31 Km NE of Catemaco, nr. Balzapote, 3-15.V.1981, C. M. & O. S. Flint; 3 males, 6 females (NMNH). Los Tuxtlas area, Los Tuxtlas Biological Station, 31 Km NE of Catemaco, Río Palma, above La Palma, 7-14.V.1981, C. M. & O. S. Flint; 7 males, 3 females (NMNH). Los Tuxtlas area, Los Tuxtlas Biological Station, 31 Km NE of Catemaco, Río Palma, below La Palma, 5.V.1981, C. M. & O. S. Flint; 7 males (NMNH). Los Tuxtlas area, Los Tuxtlas Biological Station, 31 Km NE of Catemaco, seeps at Las Cabañas, 8-15.V.1981, C. M. & O. S. Flint; 5 males, 3 females (NMNH). Los Tuxtlas area, Los Tuxtlas Biological Station, 31 Km NE of Catemaco, Río Máquinas, 4-14.V.1981, C. M. & O. S. Flint; 14 males, 2 females (NMNH). Puente Nacional, 23-24.VII.1965, Flint & Ortiz; 4 males, 5 females (NMNH). Same, but 31.VII.1966, Flint & Ortiz; 4 males, 2 females (NMNH). Pte. [Puente] Tecolapán, E Lerdo de Tejada, 4.XII.1975, C. M. & O. S. Flint; 1 male (NMNH). Río Tecolapan, Rt. 180, km 551, 25-26.VII.1966, Flint & Ortiz; 3 males, 3 females (NMNH). San Andrés Tuxtla, Estación Biológica Tropical “Los Tuxtlas”, 18°35.10'N, 95°04.50'W, ca 160 m, 17.V.2015, Kjer; 13 males, 1 female (UMSP). **GUATEMALA: El Progreso**: San Agustín Acasaguastlán, 11-21.VIII.1965, Flint & Ortiz; 4 males, 6 females (NMNH). **Jutiapa**: Laguna Nisquaya, 4.VIII.1965, Spangler; 2 males (NMNH). **Retalhuleu**: Pte. [Puente] El Niño, 16.VI.1966, Flint & Ortiz; 10 males, 6 females (NMNH). **Suchitepequez**: San Antonio de Suchitepequez, 6.VII.1965, Spangler; 1 male, 1 female (NMNH). Cuyotenango, 10-20.VI.1966, Flint & Ortiz, 3 males, 5 females (NMNH). Pte. [Puente] Ixtacapa, 18-19.VI.1966, O. S. Flint & Ortiz; 5 males, 2 females (NMNH). Fca. [Finca] Moca, 12.VI.1966, Flint & Ortiz; 7 males, 2 females (NMNH). **Zacapa**: Río Teculután; 18.VIII.1965; Flint & Ortiz; 1 male (NMNH). **HONDURAS: Choluteca**: 5 mi E Choluteca, 28.VII.1965, Spangler; 1 male (NMNH). **Valle**: Nacaome, 4.VIII.1967, Flint; 3 males, 2 females (NMNH). **EL SALVADOR: El Salvador**: Lago Ilopango, 5.VIII.1967, Flint; 2 males (NMNH). **La Libertad**: Quezaltepeque, 2.II.1965, S. S. & W. D. Duckworth; 2 males (NMNH). **NICARAGUA: Granada**: Reserva Silvestre Privada Domitila, Río cerca de Manantial, 11°42.17'N, 85°57.12'W, ca 60 m, 26.VII.2001, Chamorro & López; 17 males, 5 females (UMSP). **Jinotega**: Río El Tuma, app. 10 kms S of Santa Maura, 11°55.35'N, 86°27.80'W, 1000 m, 30.VII.2000, Chamorro & Chris; 30 males & females (alcohol). **COSTA RICA: Alajuela**: Río Pizote, ca. 5 km N Dos Ríos, 10°56.88'N, 85°17.47'W, 470 m, 9.III.1986, Holzenthal & Fasth; 1 male (UMSP). Laguna Río Cuarto & trib., 2.8 km (road) N Río Cuarto, 10°21.42'N, 84°12.90'W, 400 m, 13.II.1992, Holzenthal, Muñoz & Kjer; 4 males, 2 females (UMSP). **Cartago**: Quebrada Platanillo, ca. 5 km E Moravia de Chirripó, 09°49.27'N, 83°24.42'W, 1130 m, 6.VIII.1987, Holzenthal, Morse & Clausen, 4 males, 1 female (UMSP). **Guanacaste**: Río Tempisquito, ca. 3 km S Route 1, 10°47.40'N, 85°33.12'W, ca 70 m, 6.III.1986, Holzenthal & Fasth, 3 males, 4 females (UMSP). Parque Nacional Santa Rosa, Quebrada San Emilio, 10°51.72'N, 85°36.60'W, 300 m, 27.VI.1986, Holzenthal, Heyn & Armitage; 1 male, 1 female (UMSP). Río Góngora, sulfur mine, 4 km (air) NE Quebrada Grande, 10°53.22'N, 85°28.20'W, 590 m, 21.VII.1987, Holzenthal, Morse & Clausen; 7 males, 1 female (UMSP). Río Poza Salada, 10°47.93'N, 85°39.12'W, 10 m, 24.VII.1987, Holzenthal, Morse & Clausen; 7 males, 7 females (UMSP). Río Cuajiniquil, 10°52.87'N, 85°36.78'W, 250 m, 25.VII.1987, Holzenthal, Morse & Clausen; 6 males, 14 females (UMSP). Parque Nacional Guanacaste, Quebrada Pedregal, El Hacha, 10°58.98'N, 85°32.33'W, 300 m, 27.VII.1987, Holzenthal, Morse & Clausen; 1 male, 2 females (UMSP). **Heredia**: Estación Biológica La Selva, Quebrada El Salto, 10°25.62'N, 84°00.72'W, 50 m, 10.II.1986, Holzenthal; 9 males (UMSP). Río Puerto Viejo, 10°26.40'N, 84°00.72'W, 30 m, 10-11.II.1986, Holzenthal; 1 male (UMSP). Same, but 19.VI.1986, Holzenthal, Heyn & Armitage; 1 male (UMSP). Río Sarapiquí, 7 km W Puerto Viejo, 10°27.12'N, 84°04.02'W, 50 m, 11.II.1986, Morse & Fasth; 8 males, 1 female (UMSP). Río Bijagual, on road to Magsasay, 10°24.48'N, 84°04.57'W, 140 m, 12.II.1986, Holzenthal, Morse & Fasth; 2 males, 1 female (UMSP). Parque Nacional Braulio Carrillo, Río Peje, Est. Magsasay, 10°24.12'N, 84°03.00'W, 130 m, 25-26.VIII.1990, Holzenthal, Blahnik & Huisman; 2 males, females (UMSP). Quebrada Ceiba, 6 km E Cháves, 10°22.92'N, 83°55.32'W, 50 m, 2.VII.1992, Muñoz; 2 males, 2 females (UMSP). Río Bijagual, 3.5 km S Chilamate, 10°26.17'N, 84°03.60'W, 40 m, 1.VII.1992, Muñoz; 1 male (UMSP). **Limón**: Río Barbilla, ca. 8 km W B-Line, 10°04.02'N, 83°22.13'W, 30 m, 31.I.1986, Holzenthal, Morse & Fasth; 21 males, 25 females (UMSP). Río Telire and small trib., SE Suretka, 09°33.23'N, 82°53.52'W, ca 40 m, 1.II.1986, Holzenthal, Morse & Fasth; 2 males (UMSP). Reserva Biológica Hitoy-Cerere, Río Cerere, Est. Miramar, 09°40.27'N, 83°01.68'W, 90 m, 23-24.III.1987, Holzenthal, Hamilton & Heyn; 2 males (UMSP). Río Banano, 16 km WSW Bomba, 09°53.28'N, 83°10.02'W, 150 m, 26.III.1987, Holzenthal, Hamilton & Heyn; 3 males, 4 females (UMSP). Reserva Biológica Barbilla, Río Dantas, 15 km (rd) S Pacuarito, 09°59.63'N, 83°26.58'W, 300 m, 27-30.I.1992, Holzenthal, Muñoz & Kjer, 69 males, 42 females (UMSP). Same, but trib. to Río Dantas, 13 (km) S Pacuarito, 09°59.70'N, 83°28.62'W, 500 m, 1.II.1992, Holzenthal, Muñoz & Kjer; 8 males, 6 females (UMSP). E.A.R.T.H., Río Destierro, Pozo Azul, 10°12.48'N, 83°34.43'W, ca 10 m, 5.II.1992, Holzenthal, Muñoz & Kjer; 6 males, 6 females (UMSP). Same, but 27.VI.1992, Contreras & Muñoz; 9 males, 3 females (UMSP). Río Parismina, 10°14.88'N, 83°34.20'W, 5 m, 4.II.1992, Holzenthal, Muñoz & Kjer; 3 males, 6 females (UMSP). Río Dos Novillos, 10°13.20'N, 83°35.47'W, 20 m, 3.II.1992, Holzenthal, Muñoz & Kjer; 26 males, 34 females (UMSP). **Puntarenas**: Quebrada Pita, ca. 3 km (air) W Golfito, 08°38.52'N, 83°11.58'W, ca 10 m, 15.II.1986, Holzenthal, Morse & Fasth; 1 male (UMSP). Río Bellavista, ca. 1.5 km NW Las Alturas, 08°57.07'N, 82°50.77'W, 1400 m, 18.II.1986, Holzenthal, Morse & Fasth; 1 male, 1 female (UMSP). Reserva Biológica Carara, Río Carara, 4.3 km (rd) E Cost. Sur, 09°48.60'N, 84°34.32'W, 20 m, 12.III.1991, Holzenthal, Muñoz & Huisman, 12 males, females (UMSP). Río Jaba, 2.4 km (air) NW San Vito, 08°49.92'N, 82°59.47'W, 970 m, 13.VI.1986, Holzenthal, Heyn & Armitage; 1 male (UMSP). San Miguel, 08°52.00'N, 82°52.00'W, 14.XI.1991, Muñoz, 8 males; 2 females, 8 males (UMSP). Quebrada Bonita, 09°46.50'N, 84°36.30'W, ca 30 m, 18-20.V.1990, Holzenthal & Blahnik; 9 males, 40 females (UMSP). Same, but 11.III.1991, Holzenthal, Muñoz & Huisman; 4 males, 3 females (UMSP). Río Platanar, 6.5 km NE Buenos Aires, 09°11.70'N, 83°16.87'W, 450 m, 8-9.VII.1992, Muñoz, 12 males (UMSP). San José, Río del Sur, 1.5 km (rd) S Carara, 09°46.13'N, 84°31.87'W, 160 m, 13.III.1991, Holzenthal, Muñoz & Huisman; 3 males, 7 females (UMSP). **PANAMA: Chiriquí**: Dolega, 17.VII.1967. Flint; 1 male (NMNH). **Coclé**: El Valle, ca 820 m, 27.V.1983, Steiner; 1 male (NMNH). **VENEZUELA: Falcón**: Río Ricoa near Dos Bocas, 11°17.32'N, 69°26.07'W, ca 150 m, 8.VI.2001, Holzenthal, Blahnik, Paprocki & Cressa; 25 males, 6 females (UMSP). **Lara**: Parque Nacional Terepaima, Río Sarare nr. Sarare, 09°49.03'N, 69°11.60'W, ca 350 m, 15.VI.2001, Holzenthal, Blahnik, Paprocki & Cressa; 14 males (UMSP). **Miranda**: Río Caruao, 1.6 km S Caruao, 10°35.82'N, 66°20.77'W, 5 m, 26.I.1994, Holzenthal, Cressa & Rincón; 12 males, 14 females (UMSP). **Monagas**: Río Punceres, 09°58.93'N, 63°20.63'W, ca 80 m, 19.VII.2010, Holzenthal, Thomson & Cressa; 15 males, 4 females (UMSP). **Sucre**: Quebrada Zapateral, 1.5 km SE Las Piedras de Cocollar, 10°09.75'N, 63°47.59'W, 810 m, 9.IV.1995, Flint & Holzenthal; 1 male (NMNH), 24 males, 14 females (UMSP). Río Cocollar, 1.5 km SE Las Piedras de Cocollar, 10°09.62'N, 63°47.60'W, 810 m, 7-8.V.1995, Holzenthal & Flint; 11 males, 21 females (UMSP). **Zulia**: Caño Carichuano, 3.4 km SE Carbones del Guasare, 11°00.12'N, 72°17.10'W, 70 m, 12-13.I.1994, Holzenthal, Cressa & Rincón; 14 males, 9 females (UMSP). Los Angeles del Tucuco, 15-16.IV.1991, Menke & Hollenberg; 1 male (NMNH). Río Yasa, ca. 3 km (air) E Kasmera (Estación Biológica), 09°56.47'N, 72°43.20'W, 150 m, 14.I.1994, Holzenthal, Cressa & Rincón; 5 males, 2 females (UMSP).

*Leptonema islamarga*: **VENEZUELA: Nueva Esparta**: Isla Margarita, Asunción, Río Asunción; 02.VI.2000; Botosaneanu & Viloria; 10 males, 12 females paratypes (NBC).

## Discussion

Flint (1974a) stated that *Smicridea signata* was easily recognized by the presence of a lateral serrate process (wing-shaped process of Oláh and Johanson 2012) and a pair of apicodorsal lobes in the phallus. However, after examining the type of *Smicridea lobata*, Flint (1978) considered it and *Smicridea signata* to have nearly identical phalli, including the aforementioned processes and lobes. *Smicridea signata* and *Smicridea lobata* differ in the shape of the tenth tergum. In *Smicridea lobata* the tergites are finger-like and have a bifurcate lobe of varying sizes from the ventral margin (paraproct of Oláh and Johanson 2012) (Fig. 1), whereas in *Smicridea signata* the tergites are broader and the lobe from the ventral margin is rounded (Flint 1974a; fig. 138) (Fig. 2). Flint (1978) also mentioned that the tergites in *Smicridea lobata* were widely separated dorsomesally whereas in *Smicridea signata*, they were closer together. However, in some of the material available to us, the tergites in both species were separated roughly by the same distance. In addition to Fig. 2, the figures of *Smicridea signata* provided by Flint (1974a, figs 137–140) can be used to separate this species from *Smicridea lobata*.

Oláh and Johanson (2012) mentioned that the diagnostic character that separates *Smicridea repula* from its closest relatives, *Smicridea lobata* and *Smicridea nemtompa*, is the presence of lateral serrate processes at the mid-length of the phallus. However, as Flint (1978) noted, both *Smicridea signata* and *Smicridea lobata* also have these processes. Additionally, the illustration accompanying Oláh and Johanson’s description for *Smicridea repula* matches Ulmer’s *Smicridea lobata* illustration perfectly (Ulmer and Thienemann 1909; fig. 2). Also, the *Smicridea repula* holotype that was loaned to us fits perfectly with the examples of *Smicridea lobata* from Costa Rica, Guatemala, Mexico, Nicaragua, Panama, and Venezuela from the Smithsonian and the University of Minnesota Insect Collection. In these examples, the lateral serrate processes of the phallus vary in size, as noted by Flint (1974a) also for *Smicridea signata*. Finally, since the serrate processes of the phallus are not exclusive to *Smicridea repula*, and all the other characters between *Smicridea repula* and *Smicridea lobata* perfectly match, we consider *Smicridea repula* Oláh & Johanson, 2012 to be a junior subjective synonym of *Smicridea lobata* (Ulmer, 1909), **new synonym**.

Botosaneanu and Viloria (2002) provided a combination of characters for the inclusion of *Leptonema islamarga* in the *Leptonema davisi* species group, along with *Leptonema aterrimum* Mosely, 1933, *Leptonema davisi* Flint, McAlpine & Ross, 1987, and *Leptonema gadzux* Flint, McAlpine & Ross, 1987. However, most of the proposed characters for the inclusion of *Leptonema islamarga* in this group are rather general (e.g., small size, tibial spur formula 1/4/4, middle tibia of females not dilated, and phallus with processes), and they are not exclusive of the group, much less to the genus *Leptonema*. The authors also mentioned that the forewing pattern of *Leptonema islamarga* was extremely distinctive from other members of the genus *Leptonema*. After comparing the forewing color pattern of the paratypes (fig. 7 of Botosaneanu and Viloria 2002), and other specimens, we conclude that this forewing coloration actually corresponds to the color pattern and venation found in many species of Smicridea (Rhyacophylax). Additionally, Botosaneanu and Viloria (2002) observed a pair of gill-like appendages from the fifth sternite in both sexes. They also hypothesized that these structures replaced the raised, glandular structures of *Leptonema*. However, these structures actually correspond to the anterolateral filaments commonly present in the subgenus Rhyacophylax (Flint, 1974b). Finally, the authors recognized that the male genitalia of *Leptonema islamarga* were quite distinct from the other three species in the *Leptonema davisi* group, except for the absence of warts on the tenth abdominal tergum. The illustrations of *Leptonema islamarga* and the specimens in the type series match perfectly with the specimens we have examined of *Smicridea lobata* and with Ulmer’s illustration of *Smicridea lobata*. Accordingly, *Leptonema islamarga* Botosaneanu, 2000 is transferred to the genus Smicridea (Rhyacophylax) and placed as a junior subjective synonym of *Smicridea lobata* (Ulmer, 1909), **new combination, new synonym**.

Based on the material examined, *Smicridea lobata* is distributed in Mexico, Guatemala, Honduras, El Salvador, Nicaragua, Costa Rica, Panama, and Venezuela, and *Smicridea signata* is distributed in southwestern USA, Mexico, and Guatemala. Flint (1974a), in his redescription of *Smicridea signata* included several specimens that were actually *Smicridea lobata*. After re-examining this material, we noted that the distributions of *Smicridea lobata* and *Smicridea signata* overlap in Mexico and Guatemala. Furthermore, we observed that along with the lateral serrate processes of the phallus, the ventrolateral lobes of the tenth tergum tend to increase in size towards the southern portion of its range. However, as Flint (1974a) stated, these two species can be readily distinguished by the shape of the tenth tergum in dorsal view (Figs 1E–2). The ventrolateral lobes of the tenth tergum are bifurcate in *Smicridea lobata* and rounded in *Smicridea signata*, and the dorsomesal processes are finger-like in *Smicridea lobata* and broad in *Smicridea signata*. Additionally, the ventrolateral lobes of the tenth tergum in *Smicridea signata* present a very small spicule apically, which was not illustrated by Flint (1974a).
